# A recruitment breath manoeuvre directly after endotracheal suction improves lung function: An experimental study in pigs

**DOI:** 10.1080/03009730903177357

**Published:** 2009-09-07

**Authors:** Ihsan Kasim, Miklos Gulyas, Birgitta Almgren, Marieann Högman

**Affiliations:** ^1^Department of Pathology, Gävle HospitalGävleSweden; ^2^Centre for Research and Development, Uppsala University/County Council of GävleborgSweden; ^3^Karolinska Institute, Department of Clinical Sciences, Division of Anaesthesia and Intensive Care, Danderyd University HospitalStockholmSweden; ^4^Department of Medical Cell Biology, Integrative Physiology, Uppsala UniversityUppsalaSweden

**Keywords:** atelectasis, histology, lung, mechanical, ventilation, pigs, recruitment, suction

## Abstract

**Background:**

Atelectasis occurs after a well performed endotracheal suction. Clinical studies have shown that recruitment manoeuvres added after endotracheal suction during mechanical ventilation restore lung function. Repetitive lung over-distension is, however, harmful for the lung, and the effects of adding a larger breath, recruitment breath, directly after repeated endotracheal suction were therefore investigated.

**Methods:**

Twelve healthy anaesthetized pigs were randomized into two groups: one without and one with a recruitment breath manoeuvre (RBM), i.e. a breath 15 cmH_2_O above inspiratory pressure for 10 s during pressure-controlled ventilation. The pigs were suctioned every hour for 4 hours with an open suction system.

**Results:**

At the end of the study there was a statistically significant difference between the group given RBM and that without with respect to PaCO_2_, tidal volume (V_T_), and compliance (Crs). Without RBM, the PaCO_2_ increased from 4.6±0.4 to 6.1±1.5 kPa, V_T_ decreased from 345±39 to 247±71 mL, and Crs decreased from 28±6 to 18±5 mL/cmH_2_O. There was no change in PaCO_2_ or Crs when a RBM was given. Morphological analysis revealed no differences in aeration of apical and central lung parenchyma. In the basal lung parenchyma there were, however, greater areas with normal lung parenchyma and less atelectasis after RBM.

**Conclusions:**

Atelectasis created by endotracheal suction can be opened by inflating the lung for a short duration with low pressure, without over-distension, immediately after suction.

## Introduction

Pulmonary atelectasis occurs consistently during mechanical ventilation (1,2). Its formation may be related to different factors. Thus, atelectasis may be caused by airway closure and hence absorption atelectasis is formed (3). However, during and after anaesthesia the atelectasis may be related to the compression applied on the thorax and abdomen by a thoracic or upper abdominal procedure (4–6). Atelectasis may also occur in patients with assisted mechanical ventilation when their airway needs to be cleared of mucus (7,8). The most significant lung volume loss is associated with disconnection of the patient's circuit from the ventilator when using open suction systems (9,10). In addition, the vacuum applied during suction may result in partial collapse of pulmonary parenchyma and of small airways and thereby forming suction atelectasis (10). In a previous animal experimental model with pressure-controlled ventilation, suction decreased compliance by 27%, tidal volume by 26%, and PaO_2_ by 25%, and increased PaCO_2_ by 33% (values were obtained 10 min after suction) (11). Repeated suction, i.e. de-recruitments, might accentuate both surfactant disturbances and lung injury. In the worst case this may mean that a normal caring procedure can induce lung impairment.

The use of positive end expiratory pressure (PEEP) is mandatory in order to protect the lung from absorption and compression atelectasis during mechanical ventilation. PEEP increases lung volume and prevents collapse of small airways, which can be documented at the bedside with lung volume measurements and dynamic compliance. The setting of PEEP to prevent the lung from small airway collapse is not enough to re-expand the lung; it has to be combined with a recruitment manoeuvre (RM) (12). RM can be performed by different methods, e.g. as a vital capacity manoeuvre or as stepwise increases of PEEP (13,14). Repetitive RMs with 40 cmH_2_O were necessary to open up the lung in an animal study when recruitment was tested in a lung injury model (15). In patients with acute lung injury or acute respiratory distress syndrome (ARDS), however, there are inconsistent results from RM, and one possible reason is the inhomogeneous distribution of lung collapse/oedema (16). By giving RM to such a lung, the open areas in the lung will preferentially inflate (high compliance) rather than the targeted alveoli (areas of atelectasis). This could lead to harmful distension of the open areas of the lung. A ventilator mode with repetitive sigh has been suggested as a means to prevent absorption atelectasis (17).

Our hypothesis is that a full RM is not needed directly after suction. Instead a recruitment breath manoeuvre (RBM) should be performed as early as possible. This can prevent gas exchange impairment and regain the lung volume lost during suction, thereby preventing suction atelectasis. In clinical studies, areas of atelectasis are usually assessed by computer tomography. Histological examination by light microscopy to illustrate alveolar collapse (18), distension, or effects of intervention has been used in animal studies (15). In this study, that method was applied to verify the distribution of suction atelectasis.

## Materials and methods

### Animals

Twelve healthy anaesthetized pigs of mixed breed (Hampshire, Yorkshire, and Swedish Landrace) with a body-weight of 28.5–35 kg were investigated. The experimental protocol was examined and approved by the Ethics Committee for Animal Experiments, Uppsala, Sweden. The study was performed in accordance with recommendations of the Swedish National Board for Laboratory Animals.

### Anaesthesia

Before transport to the laboratory, the pigs were premedicated with 40 mg azoperon (Stresnil®; Janssen Pharmaceutical, Beerse, Belgium) given by intramuscular (i.m.) injection. Anaesthesia was induced with 0.5 mg atropine (Atropin®; NMPharma, Stockholm, Sweden) and a mixture of 100 mg tiltamin and 100 mg zolazepam (Zoletile® forte vet; Virbac Laboratories, Carros, France) diluted in 5 mL medetomidine (Domitor® 1 mg mL^−1^; Orion, Farmos, Finland) with a dose of 1 mL per 20 kg body-weight i.m. The animals were placed in supine position on a heating pad and intubated with a cuffed endotracheal tube, 6.0 mm inner diameter. A bolus injection intravenous (i.v.) of 0.2 mg Fentanyl® (Antigen Pharmaceuticals, Roscrea, Ireland) was given. Anaesthesia was maintained by infusion of 5 mL kg^−1^ h^−1^ of 4 g ketamine (Ketamin® Veterinaria; Zürich, Switzerland), and 1 mg Fentanyl® in 1000 mL Rehydrex with glucose (Pharmacia and Upjohn, Stockholm, Sweden). Bolus doses of 1–2 mg pancuronbromide (Pavulon®; Organon, Netherlands) were given before suction.

### Ventilation

The pigs were mechanically ventilated (Servo 900C Siemens-Elema, Solna, Sweden) in pressure-controlled mode. Since no lung injury was induced, the ventilator settings for the base-line were inspired oxygen fraction (F_I_O_2_) 0.3, PEEP 3 cmH_2_O, and a total pressure level of 12–15 cmH_2_O with a respiratory rate adjusted to achieve end-tidal CO_2_ (ETCO_2_) around 5 kPa.

### Measurements and monitoring

A catheter was inserted in the carotid artery for pressure measurements and blood sampling. A balloon thermodilution catheter was introduced in the external jugular vein and advanced to the pulmonary artery. A central venous catheter was inserted in the same vein as the thermodilution catheter. Measurements consisted of arterial blood gases (ABL 5; Radiometer, Copenhagen, Denmark), heart rate, mean arterial pressures (MAP), mean pulmonary arterial pressures (MPAP), cardiac output (CO) measured by thermodilution technique, and oxygen saturation (SpO_2_). A D-lite™ flow sensor (Datex-Ohmeda, Instrumentarium, Helsinki, Finland) was connected at the Y-piece for dynamic gas monitoring. Respiratory rate, ETCO_2_, tidal volume (V_T_), dynamic compliance (Crs), and resistance of the respiratory system (Rrs) were monitored with a CS/3 CCM™ critical care monitor (Datex-Ohmeda). The monitor was connected to a computer, and data were collected continuously (Datex-Ohmeda S/5 Collect).

### Protocols

The pigs were randomized into two groups. One group (*n*=6) was suctioned in the endotracheal tube once every hour for the period of 4 hours. In the other group (*n*=6) endotracheal suction was done in the same way but suction was followed directly by a RBM. The pressure limit was adjusted for this breath to 15 cmH_2_O above inspiratory pressure and held for 10 s. In a pre-study a pressure level to open up the lung after suction with an open suction system was found to be between 10–14 cmH_2_O in healthy pigs, and 15 cmH_2_O was therefore chosen for this study. Suction was performed with a standard vacuum device with a −14 kPa pressure. An open suction system (UNO Maersk Medical, Denmark) with a 14 French catheter was used. This catheter size was chosen to simulate the reduction in diameter with build-up of the bio-film of mucus inside the endotracheal tube with time. The catheter was inserted into the distal end of the endotracheal tube, vacuum was applied for 5 seconds, and then the catheter was removed. The disconnection from the ventilatory circuit lasted about 10 s. Supplementary oxygen was not given before or after suction. During the protocol, the ETCO_2_ was limited to reach 8 kPa, and the respiratory rate was then increased. At the end of the protocol, the lungs were removed for morphologic investigation during deep anaesthesia.

### Histological preparation

Tissue samples were taken from three different regions of the left lung, i.e. from apical (zone 1), central (zone 2), and basal (zone 3) parenchyma zones. The samples were fixed in 4% paraformaldehyde (pH 7.6) and embedded in paraffin. Blocks were cut in 4 µm sections, and the slides were stained with Mayer's haematoxylin and eosin (HE). Van Gieson staining was also performed to exclude occasional solid fibrous areas.

### Morphological analysis

Histological slides were analysed in a light microscope (Leitz DMRBE, Wetzlar, Germany) connected to a digital camera, using an image analysis program (Leica 1M 100 Image manager, Solms, Germany). Micrographs were taken from each zone. Three different areas such as normal and distended, and parenchyma with atelectasis were identified and outlined on the computer screen. Septa with bronchi and larger blood vessels, as well as fibrous tissue not regarded as belonging to the alveolar parenchyma, were outlined. The alveolar parenchyma area could be determined by marking off each area respectively. The samples were coded for the person who performed the analysis. The codes were then revealed and the ratio of normal, distended, and parenchyma with atelectasis were calculated with regard to the total parenchyma area of the section. Two samples from each zone were analysed, and a mean value for each zone was used for the statistical analysis.

### Statistical analysis

A non-parametric analysis with two related samples, the Wilcoxon signed ranks test, was used to test the statistical significance between physiological measurements at base-line and 4 h. Morphological analysis was tested within groups by the Kruskal-Wallis test and in-between groups by the Mann-Whitney U test. The Spearman rank order correlation was used for correlation analyses. All statistical calculations were made using SPSS (Chicago, USA, v. 15.0). *P*-value < 0.05 was regarded as statistically significant.

## Results

### Physiological measurements

There were no measurable changes in gas exchange or in circulation over time after repeated suction with a RBM performed directly after suction (Table I). There was, however, a minor reduction in V_T_. After suction without RBM, there were statistically significant changes in circulation, gas exchange, and ventilation. Thus, MAP, V_T_, and Crs decreased, while PaCO_2_ and ETCO_2_ increased.

**Table I.** Physiological measurement at base-line and 4 h after repeated suction with (*n*=6) or without (*n*=6) RBM. Data are expressed as mean±SD.

	Suction without RBM			Suction with RBM		
	Base-line	4 h	*P*	Base-line	4 h	*P*
Circulation						
MAP, mmHg	83±24	73±21	0.046	81±10	78±16	ns
MPAP, mmHg	21±6	23±4	ns	20±3	23±3	ns
HR, beats/min	97±11	89±15	ns	91±17	76±4	ns
CO, L/min	2.8±0.4	2.4±0.7	ns	2.6±0.8	2.2±0.2	ns
Gas exchange						
PaO_2_, kPa	19±3	18±3	ns	22±1	20±2	ns
PaCO_2_, kPa	4.6±0.4	6.1±1.5	0.046	4.7±0.5	4.9±0.4	ns
SpO_2_,%	99±1	99±1	ns	99±1	99±0	ns
ETCO_2_, kPa	5.0±0.2	6.5±1.2	0.046	5.1±0.4	5.1±0.5	ns
Ventilation						
Breaths/min	21±1	24±6	ns	20±3	19±3	ns
V_T_, mL	345±39	247±71	0.028	343±31	314±38	0.042
Crs, mL/cmH_2_O	28±6	18±5	0.028	27±3	23±2	ns
Rrs, cmH_2_O/L/s	14±2	15±3	ns	14±1	15±1	ns

RBM = recruitment breath manoeuvre; MAP = mean arterial pressures; MPAP = mean pulmonary arterial pressures; HR = heart rate; CO = cardiac output; VT = tidal volume; Crs = dynamic compliance; R_rs_=respiratory system.

### Morphology

**Figure 1.**
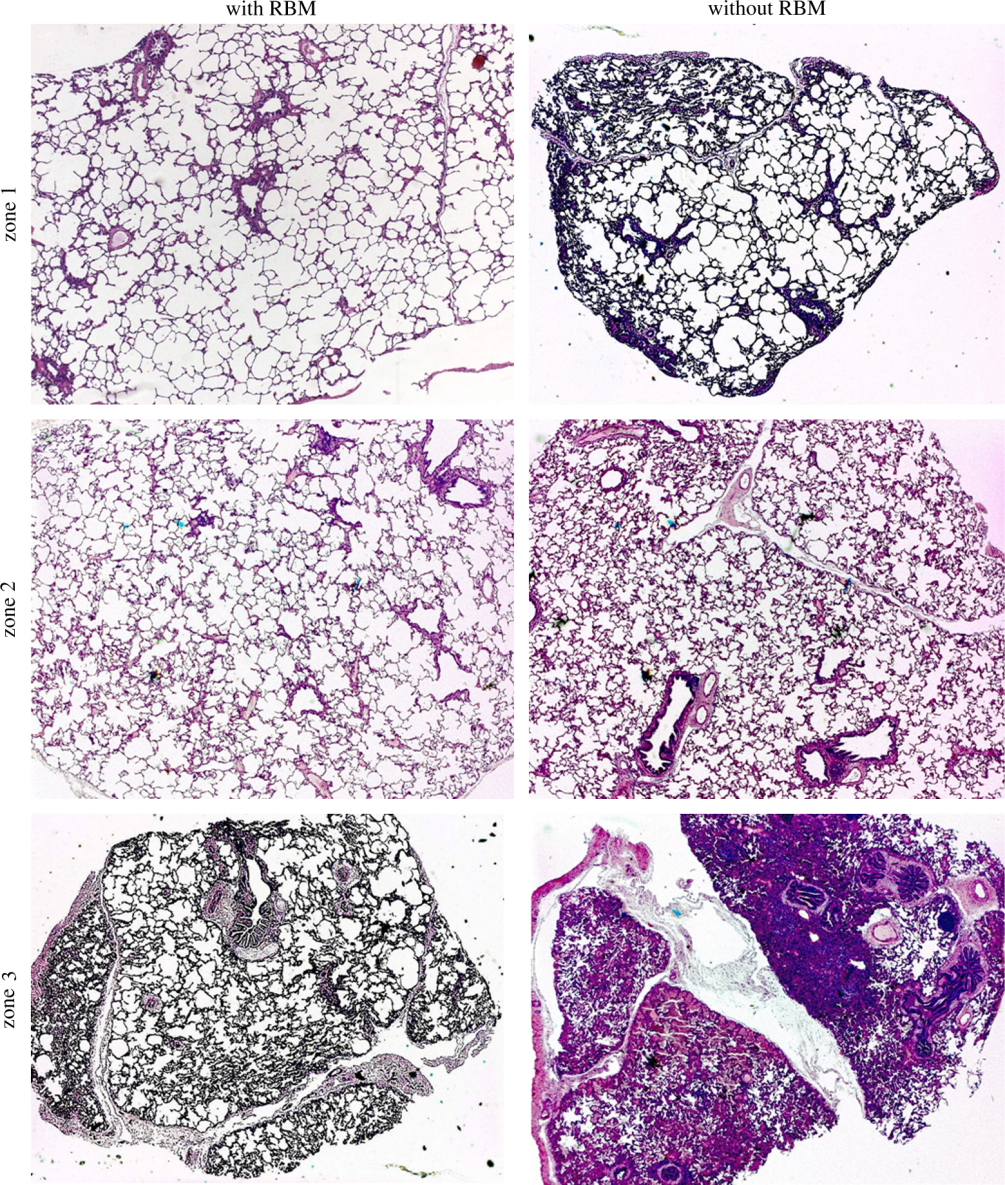
Histological comparison of different pulmonary regions with (left column) and without (right column) recruitment breath manoeuvre (RBM). Zones 1–3 are presented from the top of the picture to the bottom. Note the slightly over-distended areas in zone 1 in both treatment groups and the difference in zone 3, with less aerated areas in lung specimens from a pig not treated with RBM.

In animals not treated with RBM, areas with normal parenchyma were less abundant in zone 3 than in zones 1 and 2 (Table II, Figure 1). The areas with alveolar distension were larger in zone 1. Most importantly, areas with atelectasis were frequently observed in zone 3.

**Table II.** Morphological analysis of lung specimens at 4 h after repeated suctions with (*n*=6) or without (*n*=6) RBM. Areas in% are expressed as mean±SD.

	Suction with RBM				Suction without RBM			
	Zone 1	Zone 2	Zone 3	*P*	Zone 1	Zone 2	Zone 3	*P*
Normal	84±7	94±8	90±10	0.012	83±14	80±26	43±28	0.001
Distension	14±8	3±6	4±5	<0.001	12±8	6±5	2±5	0.014
Atelectasis	2±3	3±6	6±9	ns	5±13	14±24	55±29	<0.001

RBM = recruitment breath manoeuvre.

In the animals treated with RBM, areas with normal parenchyma predominated with the highest percentage in zone 2. Areas with alveolar distension were, however, larger in zone 1 than in the other zones. Areas with atelectasis were rare in all areas investigated.

The comparison between treatment with and without RBM showed no major pathomorphological differences between the two groups in zones 1 and 2. However, in zone 3 there was a significant difference between the two groups (*P <*0.001). There was a greater normal area (*P <*0.001) and smaller atelectasis (*P <*0.001) in the group with RBM, whereas no difference in area with distension was observed (Figure 1 and Table II).

### Correlations

For all animals investigated there was a statistically negative correlation between Crs and areas of atelectasis in zone 3 (*r*= − 0.61, *P*<0.05). There was also a sizable negative correlation between Crs and PaCO_2_ (*r*= − 0.84, *P <*0.001), and Crs and ETCO_2_ (*r*= − 0.80, *P <*0.01).

## Discussion

In this study it was found that during pressure-controlled mechanical ventilation, adding a recruitment breath directly after endotracheal suction improved the physiological performance of the lung. It hereby reopened lung tissue collapsed due to suction, which was located at the basal part of the lung, and did not over-distend already open areas.

The recruitment breath manoeuvre applied in this study clearly differs from the recruitment manoeuvres used in the concept of ‘open up the lung’ (12,19). There is a wide variety of recruitment manoeuvres, but the one to use must be chosen with regard to efficacy and risk, and that may vary depending on the patient's right heart loading status and lung properties (20). In the clinical setting the RBM should preferably be given by the same person performing the caring procedure, as the lung will easily regain its volume immediately after suction.

Morphological analysis in the group not treated with RBM showed considerable differences between different areas of the lung. The normal and distended tissue was located more apically, while the atelectasis was spread in the basal regions. With RBM exerted after suction most of the atelectasic aberrations were absent in the basal areas to the extent that they were appearing as rarely in zone 3 as in zones 1 and 2. It is worthy of note that independently of whether RBM was effective or not, distension was more abundant in the upper lobe (zone 1) than in the lower lobe (zone 3). Most likely it was caused by the mechanical ventilation and setting of PEEP (21,22). Our finding that there is no over-distension due to RBM, as can be seen in Figure 1 and Table II, leads us to the conclusion that a RBM can safely be given when considering lung volume. However, in a critically ill patient, monitoring of the haemodynamic effects is essential during RBM.

It was noticed in a recent Scandinavian survey, where 150 intensive care units (ICUs) were contacted, that less than 20% of the ICUs routinely performed recruitment manoeuvres after endotracheal suction (23).

It can be questioned if RBM should be given routinely. There is most likely a lung volume loss after a successful suction, which can easily be monitored. In the clinical setting one can monitor lung volume and lung mechanics as well as analyse arterial blood gases. A correlation between lung volume, as determined by computer tomography (CT), and Crs has been established (24). In the present study, there was a correlation between Crs and area of atelectasis, i.e. the greater the area of atelectasis, the less Crs. There was also a correlation between Crs and PaCO_2_, as well as Crs and ETCO_2_. We therefore suggest using non-invasive measurements to monitor the effects of endotracheal suction on lung function, i.e. V_T_, Crs, and ETCO_2_.

In the animals treated with suction followed by RBM, V_T_ decreased without influencing lung mechanics or gas exchange. One explanation could be that during repeated suctions lung volume was lost and the pressure level was not high enough to reopen all atelectasis which was created by suction. Another more plausible explanation is that compression/absorption atelectasis was formed, and a higher recruitment pressure was needed to expand the atelectasis. This condition has similarities with a diseased lung, where higher pressure is needed to open compression and absorption atelectasis, and this higher pressure can be harmful to the lung and also have haemodynamic effects (25). In patients with early ARDS, the addition of one sigh per minute during pressure support ventilation improved gas exchange and lung volumes (16), and therefore a recruitment breath directly after suction could possibly prevent the lung from suction atelectasis.

The limitation of the study is that we used healthy pigs, whereas most mechanically ventilated patients have a compromised lung function. The PEEP setting was 3 cmH_2_O, while for patients much higher PEEP levels are used. The results might have been different if a higher PEEP had been used, since higher PEEP has been shown to prevent loss of compliance in patients with ARDS (26). Nevertheless, we speculate that the suction atelectasis will appear after efficient suction and that the PEEP level is not enough to regain lung volume during pressure-controlled ventilation. With regard to volume-controlled ventilation, a V_T_ is most likely delivered to the open areas of the lung and will expand the fast communication lung areas resulting in over-distension. The response to suction has been shown to be different in a pig model, with more severe effects on lung mechanics and gas exchange in pressure-controlled ventilation compared to volume-controlled ventilation (11). From a pathophysiological point of view it must be better to open the area of collapse with the first breath after suction to spare the lung tissue from friction.

In conclusion, this study shows that a recruitment breath manoeuvre during pressure-controlled ventilation may improve the lung function and diminish the atelectasis created by endotracheal suction. This type of recruitment manoeuvre should not be mixed up with the recruitment manoeuvre used for regaining lung tissue that has been collapsed for hours due to absorption or compression atelectasis. Further investigations in patients are now needed to show whether this recruitment breath manoeuvre can become a clinical tool to prevent suction atelectasis.
